# Balancing Bleeding and Thrombosis: A Case of Antiplatelet Bridging with Cangrelor and Hyperfibrinolytic Disseminated Intravascular Coagulation

**DOI:** 10.1055/a-2895-4316

**Published:** 2026-07-09

**Authors:** Georg Gelbenegger, Gottfried Heinz, Robert Zilberszac

**Affiliations:** 1 Department of Clinical PharmacologyMedical University of ViennaViennaAustria; 2 Division of CardiologyDepartment of Medicine IIMedical University of ViennaViennaAustria

## 

Perioperative management of antithrombotic therapy in patients with recent coronary stent implantation requires careful balancing of thrombotic and bleeding risk. This is particularly challenging in patients requiring urgent high-risk surgery early after acute coronary syndrome, where interruption of P2Y _12_ inhibition may precipitate stent thrombosis, whereas continuation increases bleeding risk. We report a case highlighting a platelet function testing-guided cangrelor bridging strategy to maintain perioperative P2Y _12_ inhibition, followed by a fatal postoperative coagulopathy consistent with disseminated intravascular coagulation (DIC) with a hyperfibrinolytic phenotype.

A 74-year-old woman experienced in-hospital cardiac arrest due to an inferior ST-elevation myocardial infarction shortly after undergoing elective orthopedic knee surgery. Following successful resuscitation, a proximal right coronary artery occlusion was treated with drug-eluting stent (DES) implantation. Given concomitant activated protein C resistance and a history of recurrent venous thromboembolism, triple antithrombotic therapy, consisting of aspirin, ticagrelor, and unfractionated heparin, was initiated. After initial stabilization, the patient developed acute paraplegia due to an unstable spinal fracture requiring urgent surgical intervention.

To mitigate bleeding risk while maintaining an acceptable antithrombotic strategy, ticagrelor was de-escalated to clopidogrel, and aspirin was discontinued after 7 days. 1 2 3 In the early phase after percutaneous coronary intervention (PCI), thrombotic risk is markedly increased, particularly in the context of acute coronary syndrome. In this setting, temporary interruption of oral P2Y _12_ inhibition may expose patients to a critical period of insufficient platelet inhibition. To minimize this window, a platelet function testing-guided bridging strategy was implemented. Following discontinuation of clopidogrel, platelet reactivity was assessed at 12-hour intervals using multiple electrode aggregometry. 4 Intravenous cangrelor (0.75 μg/kg/min) was initiated when adenosine diphosphate (ADP)-induced platelet reactivity showed an upward trend, indicative of recovering platelet function (but before reaching the proposed cutoff value of 46 U). 5 Cangrelor was discontinued 2 h prior to surgery. This approach enabled uninterrupted P2Y _12_ inhibition until the immediate preoperative period without bleeding complications ( Fig. 1 ).

**Fig. 1:**
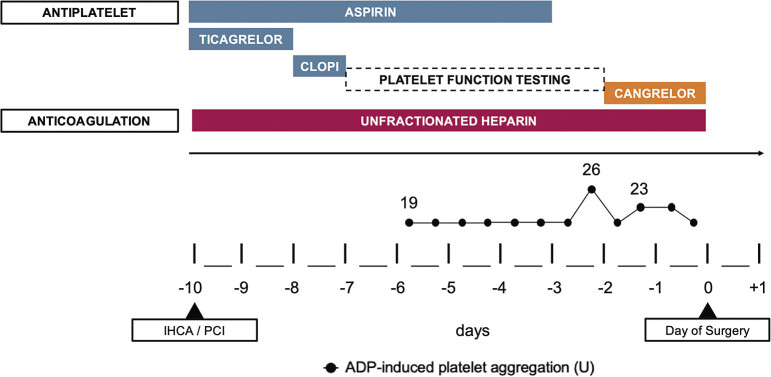
Detailed overview of platelet function testing-guided antiplatelet bridging with cangrelor. The patient received an initial loading dose of ticagrelor 180 mg followed by a 12-hourly maintenance dose of 90 mg. De-escalation from ticagrelor to clopidogrel was performed with a loading dose of clopidogrel (600 mg) given 24 h following the last dose of ticagrelor. To facilitate optimal transition from clopidogrel to cangrelor, 12-hourly platelet function testing using multiple electrode aggregometry (Multiplate analyzer, Dynabyte Medical, Munich, Germany) was performed to detect recovered platelet function and guide the initiation of cangrelor. Multiple electrode aggregometry assesses whole-blood platelet aggregation in response to specific agonists and reflects the function of circulating platelets under relatively physiological conditions. The Multiplate analyzer measures changes in electrical impedance resulting from platelet adhesion and aggregation on two independent electrode surfaces within the test cuvette. Multiplate analyzer results depend on both platelet count and function, with reduced aggregation observed at platelet counts ≤100 × 10 ^9^ /L; therefore, a platelet count >100 × 10 ^9^ /L is required for reliable interpretation (prerequisite met in our report). High on-treatment platelet reactivity, defined as >46 U by multiple electrode aggregometry using the ADP-induced platelet activation test, is associated with an increased risk of thrombotic events. In this case, cangrelor was initiated as soon as measurements indicated an upward trend of ADP-induced platelet activation. Cangrelor was given as a continuous infusion (0.75 μg/kg/min). Cangrelor was discontinued 2 h before surgery to allow for timely recovery of platelet function. Unfractionated heparin was monitored using activated partial thromboplastin time and anti-factor Xa levels (target anti-factor Xa level of 0.3 IU/mL) and discontinued 4 h before surgery. ADP, adenosine diphosphate; CLOPI, clopidogrel; IHCA, in-hospital cardiac arrest; PCI, percutaneous coronary intervention.

The management of patients with recent DES implantation who require urgent high-risk surgery presents a therapeutic dilemma between competing risks of bleeding and thrombosis. Continuous inhibition of platelet function with aspirin and a P2Y _12_ inhibitor after PCI is critical to reduce the risk of major adverse cardiovascular events and stent thrombosis. Premature discontinuation of antiplatelet therapy, especially within the first months after coronary stent implantation, is associated with a higher risk of thrombotic events (i.e., stent thrombosis). Stent thrombosis is a potentially fatal complication, occurring most frequently in the early period after stent implantation and with higher occurrence rates among patients presenting with acute coronary syndrome 6 or cardiac arrest. 7 The risk of perioperative stent thrombosis is highest during the first 4–6 weeks following PCI. Therefore, in patients who have undergone PCI with a DES within the past 3 months, the American Heart Association guidelines recommend continuation of dual antiplatelet therapy unless the bleeding risk outweighs the benefit of preventing stent thrombosis. 8 The American College of Chest Physicians guidelines acknowledge that treatment decisions should balance the bleeding risk associated with surgery under continued dual antiplatelet therapy against the thrombotic risk associated with interruption of antiplatelet therapy. 9 Thus, the need to balance ischemic protection against bleeding risk in the context of urgent noncardiac surgery after recent PCI remains a major clinical challenge, requiring individualized, multidisciplinary decision-making and, in some cases, consideration of perioperative bridging strategies.

Antiplatelet bridging implies a strategy of temporary transition from an oral to an intravenous antiplatelet agent. Although evidence supporting antiplatelet bridging before surgery is limited, it may be considered in patients deemed at high thrombotic risk who cannot safely withhold oral antiplatelet therapy and in whom a predictable interruption of platelet inhibition is required. 8 Cangrelor is an intravenous P2Y _12_ receptor antagonist that provides potent, reversible platelet inhibition with rapid onset and offset of effect. The randomized, double-blind, placebo-controlled BRIDGE (Bridging Antiplatelet Therapy With Cangrelor in Patients Undergoing Cardiac Surgery) trial investigated cangrelor as a bridging agent in patients requiring discontinuation of oral P2Y _12_ inhibitor therapy prior to coronary artery bypass graft (CABG) surgery. 10 The study showed greater platelet inhibition with cangrelor but no between-group differences in major bleeding prior to surgery and CABG-related bleeding. Ischemic endpoints were low and comparable in both groups.

The role of perioperative platelet function testing remains uncertain, and routine platelet function testing is currently not recommended. 9 Given the high thrombotic risk, we performed platelet function testing to guide antiplatelet bridging and to minimize the gap of insufficient platelet inhibition. We based our approach on proposed cutoff values for high on-treatment platelet reactivity to ADP, assessed by multiple electrode aggregometry, to guide initiation of intravenous P2Y _12_ inhibition with cangrelor. 5

## Take Home Message 1

Platelet function testing-guided antiplatelet bridging with cangrelor enabled uninterrupted P2Y _12_ inhibition without preoperative bleeding or thrombotic complications.

The immediate postoperative course was complicated by refractory shock. Transthoracic echocardiography revealed a massively dilated right ventricle with severely impaired systolic function and severe tricuspid regurgitation, highly suggestive of an acute, hemodynamically relevant pulmonary embolism. Duplex ultrasound demonstrated a long, noncompressible thrombus of the right femoral vein with echogenic material. A computed tomography scan was deemed unfeasible because of ongoing hemodynamic and respiratory compromise. Diffuse, uncontrollable bleeding ensued from mouth and nose, catheter insertion sites, and the lumbar surgical site. DIC was suspected, reflected by an increase in the DIC score from 5 on readmission to the intensive care unit to 8 within 8 h 11 ( Table 1 ). Despite massive transfusions and hemostatic support, the patient died within 24 h of surgery.

**Table 1:** Perioperative course of coagulation parameters.

	Baseline (presurgery)	ICU readmission (postsurgery)	2-h post-ICU readmission	8-h post-ICU readmission	16-h post-ICU readmission
Platelet count (150–350 g/L)	237	136	ND	36	45
Prothrombin time (80–140%)	87	57	36	35	22
Activated partial thromboplastin time (27–41 s)	51.8	>180	>180	106	74.7
Thrombin time (<21 s)	39.4	32.6	58.7	44.9	39.5
Fibrinogen (200–400 mg/dL)	749	68	<50	74	96
International normalized ratio (0.8–1.1)	1.1	1.3	1.8	1.8	2.5
D-dimer (<0.5 μg/mL)	ND	94.8	196.2	160.7	ND
DIC score	–	5	–	8	–

Abbreviations: DIC, disseminated intravascular coagulation; ICU, intensive care unit; ND, not done; ULN, upper limit of normal.

Notes: DIC score (2025):

Fibrinogen: <100 mg/dL: 1 point.

Prothrombin time prolongation: <3 s: 0 points, ≥3 s <6 s: 1 point, ≥6 s: 2 points.

Platelets: ≥100 G/L: 0 points, ≥50, <100: 1 point, <50 G/L: 2 points.

D-dimer: < ×3 ULN: 0 points, > ×3 ULN: 2 points, > ×7 ULN: 3 points.

A DIC score ≥5 points is consistent with overt DIC.

The onset of postoperative, overt DIC with both thrombotic and hemorrhagic manifestations was remarkable. In DIC, systemic activation of coagulation promotes intravascular thrombosis, whereas concomitant dysregulation of fibrinolysis may contribute to bleeding. Notably, the onset of diffuse bleeding coincided with a dramatic reduction in vasopressor requirements, suggesting partial endogenous thrombus resolution resulting in reduced right ventricular afterload and transient hemodynamic improvement, albeit at the cost of severe coagulopathy and bleeding. This interpretation is consistent with observations in other forms of hyperfibrinolytic DIC, where excessive fibrinolysis leads to profound hypofibrinogenemia and bleeding despite an underlying prothrombotic state. 12 However, the lack of viscoelastic testing (e.g., rotational thromboelastometry) limits direct assessment of fibrinolytic activity and is an important limitation.

Systemic thrombolysis is the first-line treatment for high-risk pulmonary embolism, but carries a substantial risk of major and fatal bleeding, especially in the perioperative setting. Thrombolysis was initially considered in this case, but ultimately abandoned because of the abrupt onset of uncontrollable bleeding from multiple sites. Given the contraindication to systemic thrombolysis, management focused on extensive coagulation factor replenishment and massive transfusions, including red blood cells, fresh-frozen plasma, fibrinogen, prothrombin complex concentrate, antithrombin, tranexamic acid, and vitamin K, in accordance with supportive care principles for consumptive coagulopathy. This approach is supported by the American College of Chest Physicians, which recommends individualized supportive care and correction of coagulopathy in patients with high-risk pulmonary embolism and active bleeding, rather than iatrogenic systemic thrombolysis. 13

## Take Home Message 2

Simultaneous thrombotic and hemorrhagic manifestations of overt disseminated intravascular coagulation represent a catastrophic clinical scenario with high mortality.
